# Factors controlling benzo(a)pyrene concentration in aerosols in the urbanized coastal zone. A case study: Gdynia, Poland (Southern Baltic Sea)

**DOI:** 10.1007/s11356-012-1315-0

**Published:** 2012-12-18

**Authors:** Marta Staniszewska, Bożena Graca, Magdalena Bełdowska, Dominika Saniewska

**Affiliations:** Department of Marine Chemistry and Environmental Protection, Institute of Oceanography, University of Gdansk, Al. Marszałka Piłsudskiego 46, 81-378 Gdynia, Poland

**Keywords:** Benzo(a)pyrene, Aerosols, The Gulf of Gdansk coastal zone, Gdynia

## Abstract

Annual study on the benzo(a)pyrene (BaP) concentration in aerosols in the coastal zone of the Gulf of Gdansk (southern Baltic) has been performed at Gdynia station. Combustion processes, especially domestic heating of both local and regional origin, were identified as the main sources of benzo(a)pyrene in this area. Concentrations observed during the heating season (mean 2.18 ng m^−3^) were significantly higher than these recorded in the non-heating season (mean 0.05 ng m^−3^). High benzo(a)pyrene concentrations were associated with low temperature and high humidity. Whereas high levels of precipitation usually decreased the BaP concentration in aerosols. The concentration of this factor in the studied area depended also on the wind direction and air masses trajectories. During heating season, continental air masses (coming from S, SE, SW) seemed to increase benzo(a)pyrene concentration, while maritime air masses (from N, NE, NW) caused its decrease. The differences in the BaP concentration resulting from potentially different emission levels of this compound during working and non-working days were not clearly pronounced.

## Introduction

Benzo(a)pyrene (BaP) is the major indicator of the pollution by the polycyclic aromatic hydrocarbons (PAHs). It has been identified by International Agency for Research on Cancer (IARC) as a class 1 carcinogen (i.e., carcinogenic to humans). The transformation products of BaP—i.e., peroxides, quinones, sulfur, and nitric derivatives—are also harmful to living organisms (Papageorgopoulou et al. 1999; Chetwittayachan et al. 2002).

BaP concentration in a given area is a function of anthropogenic activity and climate (Papageorgopoulou et al. 1999; Chetwittayachan et al. 2002; Ravindra et al. 2008). This compound enters the environment as a product of incomplete combustion processes. In the case of urban areas, the main sources of BaP (so-called “low emission”) are coal and wood domestic heating as well as transport-related emissions (petrol combustion, tarmac, and tire wear; Tao et al. 2007; Ravindra et al. 2008). Other significant sources of benzo(a)pyrene are heavy industry, factories, coke, and electric plants, combined heat and power plants, uncontrolled fires, and waste incineration (Ravindra et al. 2008).

After ingestion, air can be the second-most important human exposure pathway for BaP. Because of its high hydrophobicity and volatile, this compound is usually adsorbed onto particles (soot and dust) that are present in the atmosphere. In such form, BaP, as well as the other PAHs, can penetrate the respiratory system. Especially dangerous are BaP adsorbed on particles of diameters below 1–2 μm (PM1, PM2.5). They can enter pulmonary alveoli and then the particle-bound substances can reach the circulatory system and vital organs. Larger particles (PM10 and bigger) can also enter human organism, where adsorbed to them toxic compounds can be released, especially inside the gastrointestinal tract (Chetwittayachan et al. 2002; Halek et al. 2006).

In the EU countries, the quantitative measurements of BaP concentration in PM10 are performed within the frame of 2004/107/WE directive. Based on these measurements, the annual average value of BaP concentration in the air is computed. However, this information is insufficient to improve our knowledge about long and short term variability of BaP as well as the factors impacting this variability. Up to 95 % of total PAHs concentration might be associated with fine particles (<3 μm) and only a few percent of BaP is associated with aerosols larger than 11 μm (Sheu et al. 1997; Zhou et al. 2005; Ji et al. 2007; Ravindra et al. 2008). Nevertheless, significantly higher cytotoxicity (with comparable genotoxicity) of total suspended particulates fraction than the PM10 fraction in urban areas has been observed (Fabiani et al. 2008). Moreover, epidemiological studies confirmed relationships between the concentration of toxic respirable dusts and the frequency of cancer cases (i.e. lung cancer). Toxicity correlated well with the content of BaP and the other PAHs (Nielsen et al. 1996; Jung et al. 2010).

The aim of this study was to characterize the concentrations of atmospheric BaP in the urbanized coastal zone of the Gulf of Gdansk and identification of major factors controlling the variability of BaP concentrations in such kind of environments.

## Studied area

**Fig. 1:**
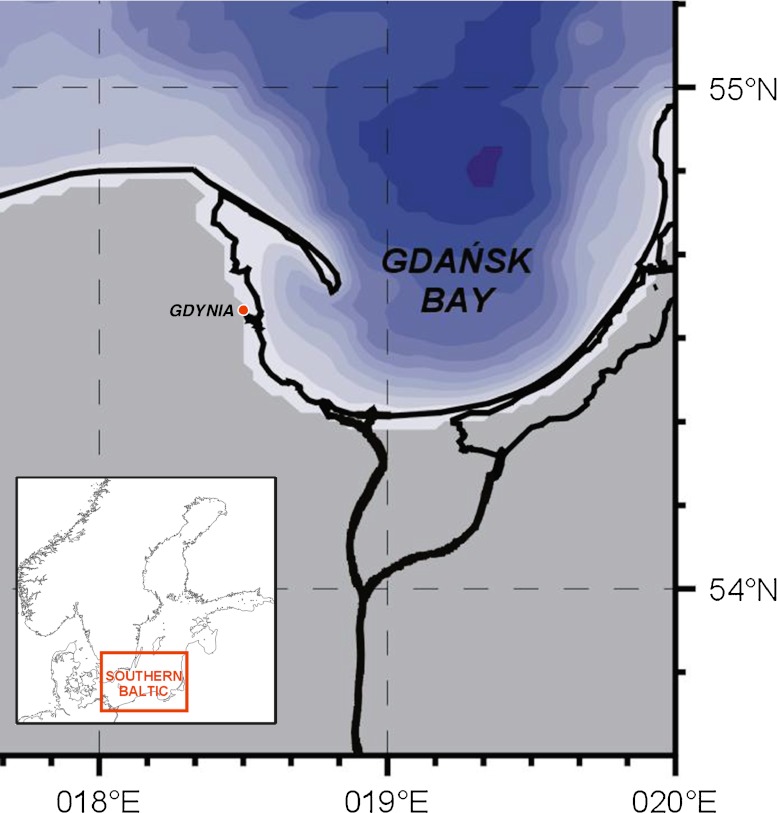
Location of Gdynia (the sampling place)

Gdynia, where the investigation was performed, is a harbor city with significant tourism and recreational activities located at the coast of the Gulf of Gdansk (Baltic Sea, Poland; Fig. 1). Together with Sopot and Gdansk, it forms the metropolitan agglomeration called Tricity. The area of Gdynia equals 135 km^2^ and its population is 250,000 (Statistical Yearbook 2008).

Potential sources of BaP in Gdynia are power and heating plants, the industry, incineration plants for oily and hospital wastes as well as paint pyrolysis plants. The level of the air pollution in Tricity is also influenced by the households that use domestic coal heating (ca. 8 %) and intensive car traffic (Statistical yearbook 2008).

## Material and methods

Aerosol samples were taken between 17.XII.2007 and 17.XII.2008 in the centre of Gdynia, ca. 500 m from the coast, at the height of 30 m (on the roof of the Institute of Oceanography Building). Aerosols were collected on ignited glass fiber filters (pore size of 0.4 μm), placed in a Teflon holder connected to a vacuum pump with the mean flow rate of 0.8 m^3^·h^−1^. Filters were changed under a laminar flow hood thrice a week - on Monday (sampling time from Friday to Monday), Wednesday (sampling time from Monday to Wednesday), and Friday (sampling time from Wednesday to Friday). The samples were kept frozen until the analysis (−20 °C).

The isolation of BaP from the collected samples was conducted by means of solvent extraction (4 cm^3^ acetonitrile: dichloromethane 3:1 *v*/*v*) in an ultrasonic bath (time, 10 min; extraction temperature 21 °C; Periera et al. 2001; Halek et al. 2006).

BaP concentration was determined with the use of liquid chromatography with a fluorescence detector (excitation, *λ* = 296 nm; emission, *λ* = 408 nm). The chromatographic separation was performed using Nucleosil column (100-5 C18 PAH, 250 mm/4.6 μm) and mobile phase gradient acetonitrile: water. The recovery of BaP in reference to the certified material (SRM-2585) amounted to 83 %. The detection limit of the method was determined to be 0.01 ng∙m^−3^.

During this study the meteorological conditions (T—air temperature, V—wind speed, B—wind direction, R—amount of precipitation, W—relative humidity, and P—atmospheric pressure) were recorded continuously by the HUGER WEATHER STATION. Forty-eight-hour air-mass backward trajectories at 6 h intervals, at 20, 500, and 1,000 m above the starting point located at 20 m a.g.l., were calculated by NOAA HYSPLIT MODEL were taken from GDAS Meteorological data (http://www.arl.noaa.gov/ready.html).

Statistical analysis was performed using Statistica 8.0 software. Apart from basic statistics, Mann–Whitney *U* tests, Kruskal–Wallis ANOVA, principal components analysis, and cluster and regression analyses were used. The significance level (*p*) in the hypothesis tests for all analyses was < 0.05.

## Results and discussion

### Meteorological conditions

Meteorological conditions characterizing the sampling period (17.XII.2007–17.XII.2008) are presented in Table 1. Average annual temperature in Gdynia in 2008 was +10.6 °C. It means that the year 2008 was one of warmest years in this decade, what resulted in shorter than average heating season (Szymanska et al. 2008). The coldest was December 2008 whereas the warmest was July 2008. The lowest relative humidity was recorded in spring/summer (May/June) 2008, whereas the highest values were observed in winter. Maximum wind speeds were noted in winter, especially in February 2008 (>4 m·s^−1^). In most of the cases (91 % of data), precipitation did not exceed 13.2 mm. Relatively high precipitation (28.6–92 mm) occurred between December 2007 and May 2008 as well as in November 2008.

**Table 1:** Meteorological condition during measurements performed in Gdynia (17.XII.2007–17.XII.2008)

Variable	Average	Min.	Max.
Air temperature (°C)	10.6	−4.3	23.1
Wind speed (m·s^−1^)	2.2	0.0	5.1
Amount of precipitation (mm)	5.2	0.0	92.5
Relative humidity (%)	71.8	45.1	93.4
Atmospheric pressure (mbar)	1,009	981	1,034

### Benzo(a)pyrene concentration in aerosols

**Fig. 2:**
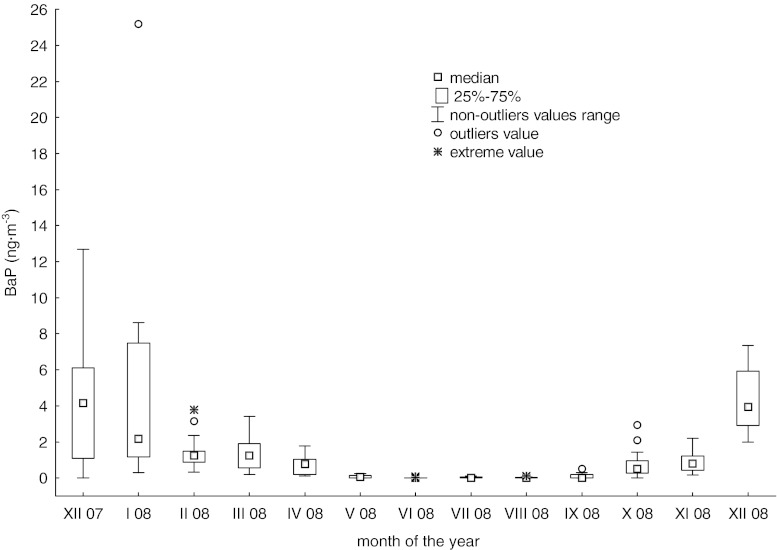
Benzo(a)pyrene concentrations in aerosols in Gdynia in subsequent months of the sampling period (17.XII.2007–17.XII.2008)

An annual average concentration of benzo(a)pyrene in aerosols in the studied area exceeded the value (1 ng∙m^−3^) acceptable for the EU countries (Directive 2004/107/WE; Table 2). At the same time, a clear seasonality (Table 2) and large intra-monthly variability (Fig. 2) of benzo(a)pyrene concentration were observed. During the heating season (17.XII.07–30.IV.08; 1.X.08–17.XII.08) both average and median concentrations clearly exceeded the values observed in the non-heating season (1.V.08–30.IX.08) (Table 2). Over the most of the heating season, the benzo(a)pyrene concentration was higher than 1 ng∙m^−3^. But actually there is no reference point to our results. The ambient air quality standards determine only the value of the acceptable annual average benzo(a)pyrene concentration. The utility of such an indicator seems to be questionable considering continuous exposure to high concentrations within a few months of the year. Therefore, determination of the acceptable daily or monthly average concentration of benzo(a)pyrene would be very valuable.

**Table 2:** Basic statistics of BaP concentrations (ng∙m^−3^) in aerosols during measurements performed in Gdynia (17.XII.2007–17.XII.2008)

Variable	Sampling period				
	(17.XII.07–17.XII.08)	(17.XII.07–30.IV.08; 1.X.08–17.XII.08) heating season	(1.V.08–30.IX.08) non-heating season	Weekends	Workdays
Number of measurements	153	89	64	58	94
Average concentrations ± standard deviation	1.29 ± 2.75	2.18 ± 3.33	0.05 ± 0.09	1.94 ± 4.72	0.91 ± 1.51
Min.-max.	n.d.− 25.20	0.11–25.20	n.d.−0.36	n.d.− 25.20	n.d.− 8.26
Median concentrations	0.34	1.10	< LOD	0.52	0.30
(Lower–upper quartile)	< LOD− 1.25	0.52–2.36	< LOD− 0.07	0.06–2.05	< LOD− 1.22

*LOD* limit of detection

Some difficulty in comparison of the obtained results with other studies is the fact that mostly they report the average values. However, positive skewness of results distribution and the outlying values are likely to lead to an overestimation of averages. In presented study 93 % of BaP concentration level did not exceed 4 ng·m^−3^, but some very high values were recorded on weekends prior to Christmas (12.68 ng·m^−3^) and on the New Year’s Day (25.02 ng·m^−3^). Such a distribution of data causes a strongly overestimation of the average BaP concentration and also influences the results of the statistical methods. In result, the obtained mean BaP concentration was about three times higher than that reported for study area by Czempińska and Zarembski (2008). At the same time, such average BaP concentration in aerosols as obtained in this study is still lower than that reported by various authors for this and other areas in Poland (Table 3). Relatively low concentrations of analyzed compound resulted probably from low industrialization of the studied area, relatively small number of ‘low emission sources’ (in comparison to other Polish cities) and the influence of maritime (“clean”) air masses. Concentrations of BaP lower than these measured in Gdynia were observed in a non-urbanized woody area “Puszcza Borecka” (Table 3). In the other European countries, in strongly urbanized areas (Italy, Hungary) or areas with intensive coal and wood domestic heating (Czech Republic), the average atmospheric BaP concentrations are comparable or higher than these observed in Gdynia (Table 3).

**Table 3:** The average concentrations of BaP (ng∙m^−3^) in aerosols in selected areas

	City	BaP (ng∙m^−3^)	Particle size	Remarks	Reference
POLAND	Gdansk-Przeróbka	3.8/0.1^a^	PM10	North Poland (coast of Baltic Sea)	Report WSSE (2007)
	Gdynia	2.18/0.05^a^ 1.29 (average from 17.XII.2007 to 17.XII.2008)	TSP		This work
	Gdynia	0.8 (average annual)	PM10		Czempińska and Zarembski (2008)
	Wejherowo	12.1/0.9^a^	PM10	North Poland (inland)	Report WSSE (2007)
	Kościerzyna	9.3/0.5^a^	PM10		
	Słupsk	7.3/0.6^a^	PM10		
	Chorzów	32.3/0.8^a^	PM10	South Poland (industrial field)	Report WSSE (2005)
	Gliwice	28.6/0.1^a^	PM10		
	Katowice	20.1/1.2^a^	PM10		
	Tychy	36.9/1.5^a^	PM10		
	Puszcza Borecka	1.14/0.05^a^	PM10	Non-urbanized woody areas (north-east Poland)	Bogucka (2009)
OTHER COUNTRIES OF THE EU	Prachatice/Teplice (Czech Republic)	5.50/0.51^a^	TSP	Health-resort	Lenicek et al. (1997)
	Taranto (Italy)	0.03–65.62/0.033–3.55^a^	PM10	Urbanized areas	Filippo et al. (2010)
	Keszthely, Tihany, Siofok (Hungary)	2.56/0.18^a^	TSP	Non-urbanized (north part of the Lake Balaton)	Bodnar and Hlavay (2005)

^a^Heating/non-heating season

It should be noted that some BaP concentration, which are presented in Table 3, are referred to PM10. Kubica (2003) suggested that the contribution of particles smaller than 10 μm in TSP originating from coal and wood combustion amounts to ca. 70 %. According to Lewandowska et al. (2010), the contribution of PM10 to TSP in Gdynia ranged from 35 % to 92 %. In addition, most of PAHs concentration might be associated with fine particles (see Intoduction). In a result, concentration of BaP in PM10 is probable equal or smaller then in TPS.

### Factors controlling the variability of benzo(a)pyrene concentration in aerosols

#### Meteorological conditions

**Fig. 3:**
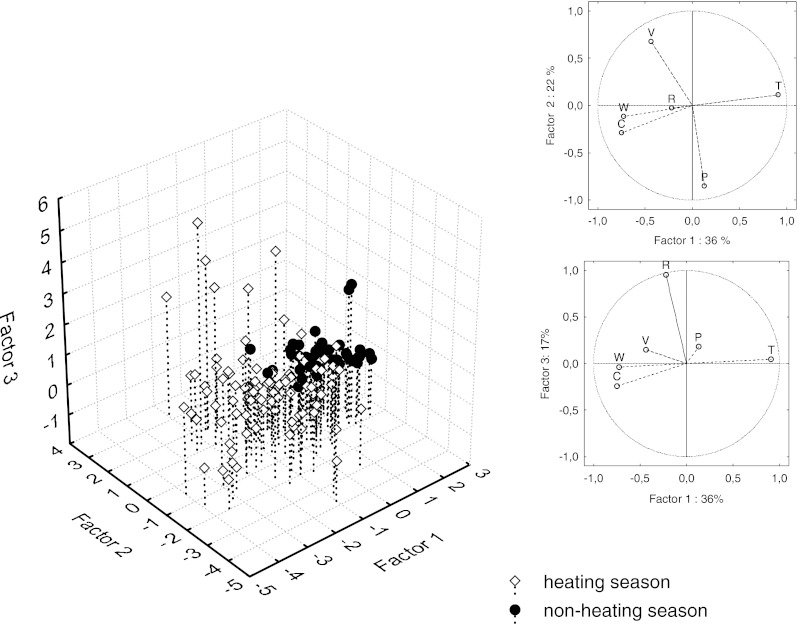
Factor scores for individual cases of the dataset containing meteorological conditions (*V* wind velocity, *R* amount of precipitation, *T* air temperature, *P* atmospheric pressure, *W* relative humidity, *C* BaP concentration) and benzo(a)pyrene concentration (*C* BaP concentration) in aerosols in Gdynia (excluding Christmas and New Year’s Eve)

**Fig. 4:**
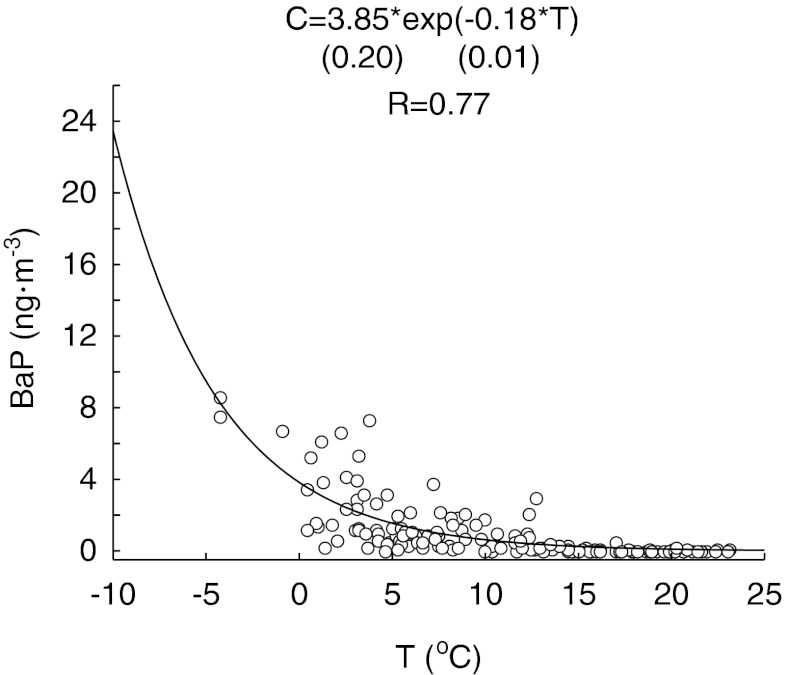
Relationship between the air temperature (*T*) and benzo(a)pyrene concentration (*C*) in aerosols in Gdynia (excluding concentrations of BaP during Christmas and on the New Year’s Eve). Standard errors of regression coefficients are given in the *brackets* (both coefficients were statistically significant, *p* = 0.00)

Depending on natural climate conditions different meteorological factors tend to control the BP concentrations in aerosols (Papageorgopoulou et al. 1999; Chetwittayachan et al. 2002; Callen et al. 2008). The principal component analysis of measured meteorological parameters and BaP concentrations was performed. It distinguished three factors explaining 74 % of data variability. Factor 1, controlling 36 % of observed variability, was correlated with temperature, relative humidity and benzo(a)pyrene concentration. Factor 2 explained 22 % of the variability and was strongly influenced by the wind speed and atmospheric pressure. Factor 3, related to the amount of precipitation, explained 17 % of variability of the dataset (Table 4). Factor 1 quite clearly separated the concentrations of benzo(a)pyrene measured in the heating and non-heating seasons (Fig. 3). High temperatures, typical for non-heating season, corresponded to low BaP concentrations, while low temperature, characteristic for heating seasons, corresponded to high BaP concentrations. The only exceptions (i.e., high concentration of BaP and high temperature) were observed when relatively high air temperatures (>10 °C) occurred during the heating season. Such a situation happened in October 2008. High BaP concentrations in heating season probably resulted from an increase in the pollution from domestic heating and 4–10-fold higher emission of exhaust fumes from traffic in winter (Kiss et al. 2001). Such high emission of exhaust fumes is caused by higher fuel consumption in low air temperatures. Furthermore, coal and wood combustion is a source of soot and fly ash which strongly adsorp BaP. In such form, BaP is more resistant for photooxidation (Ravindra et al. 2008). Low air temperature favors increased benzo(a)pyrene emission to the atmosphere not only in a direct way (heating), but also decreases the vapor pressure of BaP, what increases its affinity to aerosols (Ravindra et al. 2008). Moreover, the low air temperature in winter typically causes lowering of the mixing height what results in increased concentrations of BaP in the lower layers of the atmosphere. In many studies, thermal degradation caused by solar radiation was also reported to have a significant influence on the BaP removal from the atmosphere in warm months (Chetwittayachan et al. 2002; Tham et al. 2008; Callen et al. 2008). An increase in the concentration of ^•^O_3_ and ^•^OH and ^•^NO_2_ radicals also favors chemical transformations of all PAHs into other compounds, i.e. nitric derivatives (Papageorgopoulou et al. 1999; Park et al. 2002). According to Ravindra et al. (2008), half life time of BaP under laboratory conditions (simulated sun light) equaled 5.3 h, while it was ten times shorter after the addition of ozone, regardless of the irradiance conditions. In a result of the complex nature of processes influencing BaP concentration in the air, exponentially increased of this constituent with the decrease in the air temperature was observed (Fig. 4).

**Table 4:** Principal component analysis (PCA) factor loadings for meteorological conditions and benzo(a)pyrene concentration in aerosols in Gdynia (17.XII.2007–17.XII.2008)

Variable	Factor 1	Factor 2	Factor 3
Amount of precipitation (mm)	−0.23	−0.02	**0.95**
Wind speed (m·s^−1^)	−0.44	**0.68**	0.14
Relative humidity (%)	**−0.72**	−0.11	−0.05
Atmospheric pressure (mbar)	0.13	**−0.85**	0.18
Air temperature (°C)	**0.91**	0.11	0.04
BaP (ng∙m^−3^)	**−0.75**	−0.29	−0.24

**Fig. 5:**
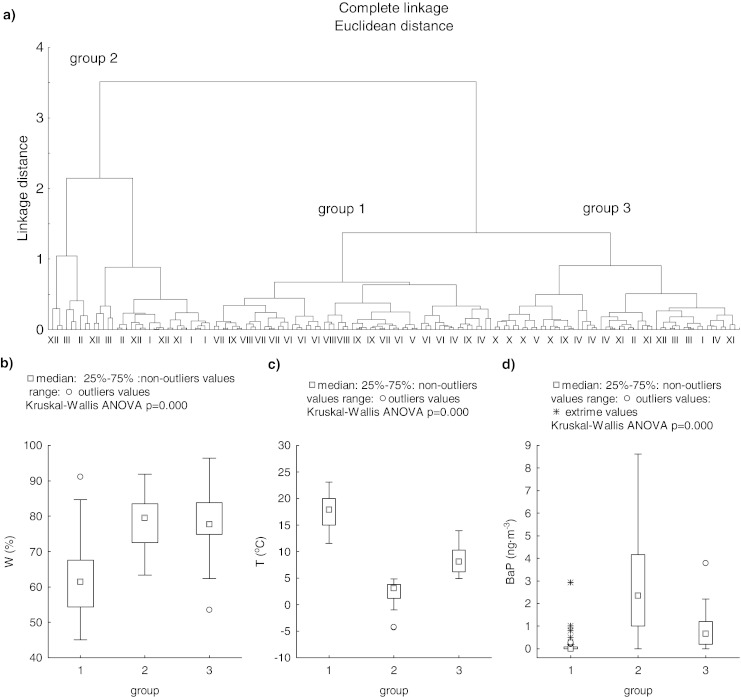
Cluster analysis results **a** with regard to the air temperature and relative humidity in Gdynia (17.XII.2007–17.XII.2008), as well as **b** benzo(a)pyrene concentrations, **c** temperature, and **d** humidity in groups distinguished by the analysis

The principal component analysis indicated strong correlation between the air temperature, relative humidity and benzo(a)pyrene concentrations (Table 4). Many studies (Park et al. 2002; Mantis et al. 2005; Callen et al. 2008; Tham et al. 2008) point at relative humidity, beside temperature, as a factor controlling particulate bound PAHs concentration in temperate zones. In order to investigate the influence of both temperature and relative humidity on the benzo(a)pyrene concentrations in the studied area, the cluster analysis was employed. This is an exploratory data analysis tool which encompasses a number of different algorithms and methods for grouping objects of similar kind into respective categories. In other words, cluster analysis divides data into groups (clusters) so that the objects in the same cluster are more similar to each other than to those in other clusters. Thus, cluster analysis discovers structures in data without explaining why they exist (Stanisz 2007; http://www.statsoft.com/textbook/cluster-analysis/). These techniques have been applied to a wide variety of research problems. Hartigan (1975) provides an excellent summary of the many published studies reporting the results of cluster analyses. In the case of presented data, the cluster analysis with regard to the air temperature and relative humidity has been performed. It distinguished three groups (Fig. 5a). After identification which data belong to each groups, significant differences between them have been found (Kruskal–Wallis ANOVA; Fig. 5b, c). Also the concentrations of BaP (Fig. 5d) in these groups were significantly different (Kruskall–Wallis ANOVA; Fig.5d). Relatively high concentration of this parameter was noted in groups 3 and 2 which were characterized by high humidity and low temperature (Fig. 5b, c). Many aforementioned interplaying processes favor high concentrations of BaP during low air temperature periods. In the case of humidity, higher values of this parameter favors the creation of larger aggregates, which are not transported over large distances, and in majority contribute to the air pollution close to an emitter (Chetwittayachan et al. 2002; Bełdowska et al. 2012; Ravindra et al. 2008). Moreover, low temperature together with high humidity cause a decrease in the wind chill temperature, what can result in more intensive residential heating and therefore increased BaP emission.

**Fig. 6:**
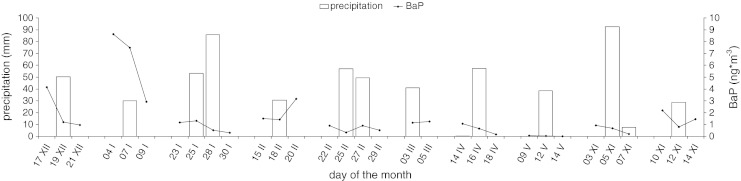
Concentration of BaP and the amount of precipitation in selected cases (samples taken before, during and after an intense precipitation event)

The studies conducted in tropical regions (Thornhill et al. 2008) indicated the major influence of precipitation on the BaP concentration. High levels of precipitation causing particle removal by washout are one of the factors responsible for reducing the ambient BaP concentration. In the studied area precipitation higher than 28.6 mm in most cases caused the decrease in the BaP concentration in samples collected during and/or after the rainfall event (Fig. 6).

#### Air masses backward trajectory

During the heating season, the results of the backward trajectory analysis were consistent with the known location of the BaP source. The continental air masses (S, SE, SW sectors) were affected by the heating contaminations and therefore characterized by higher average BaP levels (2.86 ng∙m^−3^) in comparison to these observed in the maritime air masses (N, NE, NW sectors) for which the average BaP concentration was 1.27 ng∙m^−3^.

**Fig. 7:**
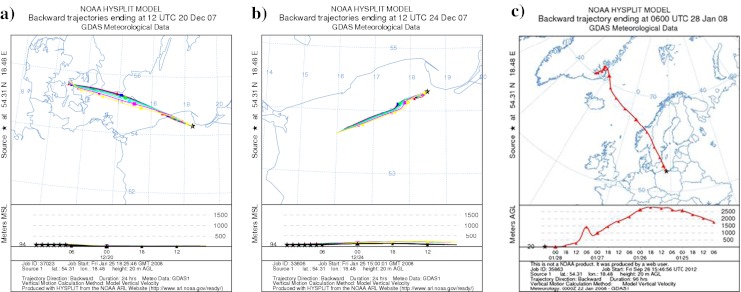
Change of the backward trajectories of air masses during the time when low (**a** 20–21.XII.2007; **c** 28–30.I.2008) and high (**b** 22–24.XII.2007) BaP concentrations were observed

The impact of the air masses trajectory on the BaP concentration was particularly visible during periods of high concentration of this component in December 2007–January 2008. When the trajectory of the air masses changed from NE (Fig. 7a) to SW (Fig. 7b) and the air was coming from over the land on 22–24.XII.2007, the BaP concentration raised from 0.99 to 12.68 ng∙m^−3^ and remained elevated to 25.I.2008 (up to 25.20 ng∙m^−3^). The mean wind speed in mentioned period of high BaP concentration (22.XII.2007–25.I.2008) was changeable (from 0 to 5 m∙s^−1^) suggesting both local and regional sources of pollution. At the end of January 2008 the concentrations of BaP decreased again below 0.50 ng∙m^−3^. It resulted from the influence of the incoming “clean” maritime air masses of high wind speed (3–5 m∙s^−1^; Fig. 7c). In addition, the daily average temperatures during the periods of high BaP concentration were lower (rang from −4 °C to +6 °C) compared to the temperature during the period of small BaP concentrations (range from +3 °C to +6 °C). Consequently, the observed variability of BaP content could be partly connected to the fluctuation of the temperature.

During non-heating season, the cleansing effect of the maritime air masses on atmosphere pollution has not been observed. This may suggest that domestic heating significantly contributed to the atmosphere pollution by BaP at the sampling site.

#### Short-time variability of benzo(a)pyrene concentration of aerosols

In urban areas high automobile traffic potentially enhances the emission of BaP during working days compared to weekends. In turn, in the heating season higher emission might be expected when people stay at home longer as a result of more intensive residential combustion. In the studied area, the differences between the benzo(a)pyrene concentrations in aerosols on working and non-working days were investigated in three ways - for the whole set of data, as well as for heating and non-heating season separately. The analysis of the entire data set revealed that the BaP levels were approximately twice higher on non-working days, however, the difference was not statistically significant (Mann–Whitney *U* test, *p* = 0.19; Table 2). Significant differences between BaP concentrations on working and non-working days were observed during the heating season (Mann–Whitney *U* test, *p* = 0.04). However, the observed differences were caused mostly by very high benzo(a)pyrene concentrations measured in weekends prior to Christmas 2007–12.68 ng∙m^−3^ and in New Year 2007/2008–25.02 ng∙m^−3^. After excluding these values, the differences were not statistically significant (*U* test, *p* = 0.11). Considerable diversity in the BaP concentrations noted during working and non-working days was observed also in non-heating season (Mann–Whitney *U* test, *p* = 0.04). Weekends at this season were characterized by higher frequency of large BaP concentrations compared to these noted on working days. Such a trend results from including May and September into the non-heating season. The air temperature during these months can be relatively low which in some cases causes enhanced domestic heating. During the study period 75 % of the temperature values measured in May and September were below 16 °C. After excluding May and September from the non-heating season, the differences between working and non-working days became insignificant (Mann–Whitney *U* test, *p* = 0.22).

## Conclusions

The average annual concentrations of BaP in aerosols in the urbanized coastal zone of the Gulf of Gdansk (1.29 ng∙m^−3^) were lower than these observed in other urbanized and affected by intensive coal and wood domestic heating areas in Poland and in European countries. At the same time the obtained concentration exceeded the acceptable for EU countries (Directive 2004/107/WE) annual average value (1 ng∙m^−3^).

Combustion processes, especially domestic heating of both local and regional origin, were identified as the main sources of benzo(a)pyrene in study area. As a result, BaP concentrations during the heating season were significantly higher than those observed in the non-heating season. Strong seasonal variability suggests that the establishing of monthly acceptable level of concentration of benzo(a)pyrene would be valuable.

BaP concentrations in aerosols in the studied area are clearly dependent on the air temperature and relative humidity. Low temperature and large relative humidity favor high concentration of BaP in aerosols, whereas precipitation higher than 28.6 mm usually decreases the BaP concentration in aerosols.

Wind direction and air masses trajectories play a major role in determining the BaP concentrations in aerosols in the studied area during the heating season. Continental air masses (S, SE, and SW) increase the BaP concentrations, while maritime air masses coming from over the Baltic Sea (N, NE, NW) cause the decrease in the concentration of this compound.

The differences in the BaP concentration resulting from potentially different emission levels of this compound during working and non-working days were not clearly pronounced. For the non-heating season it results from the artificial division of the study period into heating and non-heating season. Whereas for the heating season it is caused by some incidental very high BaP concentrations noted at the turn of December and January as well as during the Christmas season. This problem requires further study.
